# Influence of static lumbar flexion on the trunk muscles' response to sudden arm movements

**DOI:** 10.1186/1746-1340-13-23

**Published:** 2005-11-23

**Authors:** Gregory J Lehman, Stephen Story, Robert Mabee

**Affiliations:** 1 Department of Graduate Studies, Canadian Memorial Chiropractic College, Toronto, ON, Canada; 2 Undergraduate Department, CMCC, Toronto, ON, Canada

**Keywords:** EMG, spine stability, trunk muscles, creep, feedforward

## Abstract

**Background:**

Viscoelastic creep of lumbar ligaments (prolonged forward bend) has been shown to negatively influence the spine's muscular reflexive behaviour and spinal stability. No studies to date have investigated the influence of spinall viscoelastic creep on the feedforward response of the trunk muscles to sudden arm raises.

**Methods:**

Surface myoelectric activity was collected from the transversus abdominis/internal oblique, the lower erector spinae and the deltoid muscle during sudden ballistic arm raising before and after 10 minutes of prolonged forward bend in 11 healthy participants free of low back injury. The timing of trunk muscle activity relative to the deltoid muscle was calculated for 5 trials before and 5 trials after the creep procedure.

**Results:**

Viscoelastic creep had no influence on the feedforward response of the trunk muscles during sudden arm raises. A feedforward response of the trunk muscles was not seen in every study participant and during every trial.

**Conclusion:**

Passive trunk muscle fatigue does not appear to influence the timing of the stabilizing role of the investigated trunk muscles to sudden arm flexion.

## Background

Maintaining adequate spinal stability is considered necessary in the avoidance of low back injury [1]. Spinal stability is theorized by Panjabi [2] to be maintained by the interaction of three systems: the active system, the passive system and the neural control system. The active system is composed of the muscles and related tendons, the passive system consists of the ligaments, discs and structural anatomy, while the neural system coordinates the interaction between the active and passive system via proprioceptive feedback and feed-forward input in response to challenges to spinal stability. The role of the three systems is currently being delineated via human and animal experiments.

When the muscular elements are removed from the spinal column it will buckle under a compressive load as low as 2 kg. Therefore, trunk muscles are necessary for providing spinal stability through out the spine's range of motion and particularly when the spine is in its neutral zone. In the neutral zone the passive ligaments provide little resistance to movement and therefore provide little stability. Muscles function much like guy wires on a ship's mast. The greater the tension in the guy wires and the greater the number of guy wires, the greater the stability and load supported by the mast. Adequate muscular activation is therefore necessary to achieve joint stability. Cholewicki et al [3] demonstrated that at a neutral spinal posture trunk muscles show a co-activation level of 1.7% of their maximum activity (MVC). When the spine is loaded (32 kg) the stability demands increase and muscle co-activation increases to 2.9% of MVC. Patients with low back injury have been shown to have higher trunk muscle co-activation possibly as a response to stabilize their relatively unstable spines compared with a pain free population [4].

The work of Solomonow and colleagues has shown that ligaments appear to function more as proprioceptive feedback than as structural supporting elements [5,6].

Solomonow et al [5] have demonstrated that loading and stimulation of the supraspinal ligaments in a feline model and humans during surgery results in reflexive muscle activation of the multifidus muscle. This reflexive activation suggests that the multifidus is responding to a change in stability (the strain of the ligament) by increasing its activation and in turn attempting to stabilize the spine. This reflexive muscle activation relationship has also been demonstrated between the discs and the multifidus [7] and the SI joint and the gluteus maximus, multfidus and longissimus [8].

External factors may influence the stability of the spine and the interaction between the three systems. Fatigue, vibration and low back pain have been shown to increase the time between sudden loading of the spine (the spine is either abruptly bent forward or backward or perturbed in some manner) and the trunk muscle's response to this perturbation in any attempt to stabilize the spinal system [9,10]. This increased latency response suggests that the spine may become unstable because of external factors.

Viscoelastic creep of the supraspinal ligament due to repetitive flexion has also been shown to compromise spinal stability. In a feline model repetitive static and dynamic flexion (resulting in creep of the ligaments) has resulted in; an attenuation of the protective reflexive response of the multifidus, muscle spasm and a delayed hyperexcitability of trunk muscles [11-13]. The human equivalent of these feline experiments have occurred with participants holding a forward bend posture for more than 10 minutes to induce viscoelastic creep of the lumbar spinal tissues. Solomonow [14] investigated the influence of static and repetitive forward bending on the flexion-relaxation phenomenon (FRP) during forward bending. The flexion relaxation phenomonen is the occurrence of muscle activity silence during a small range of motion around peak forward bending. It is believed that at peak flexion the flexor Moment created by forward bending is resisted by the passive tissues rather than active muscular contraction [15]. Following static lumbar flexion Solomonow [14] found that the creep developed in the viscoelastic structures of the spine caused the erector spinae muscles to remain active longer during anterior flexion and to become active earlier during extension. Granata et al [16] investigated the influence of viscoelastic creep of the lumbar spine on the trunk muscle's reflexive response to sudden loading. Explained simply, this experimental procedure sees a participant suddenly pulled forward. The trunk muscles respond reflexively to the forward displacement of the trunk and become reflexively active to stabilize the spine and volitionally active to return the spine to its neutral position. Experimenters measured the time that the trunk motion occurred, how much force was applied to cause the perturbation and the resulting onset time and amplitude of the EMG signals of the trunk muscles to calculate the timing of reflexive trunk muscle activation as well as the reflexive gain of the trunk muscles. Granata [16] found that following 15 minutes of lumbar viscoelastic creep there was a tendency toward an increase in the gain of the reflexive response but no change in reflex onset latency.

The transverse abdominis has been shown to be an important muscle in providing stability to the lumbar spine and the sacroiliac joint [17,18]. In addition to providing stability to the sacroiliac joint via a force closure mechanism[18] the transverse abdominis appears to act in an anticipatory feedforward manner to self-induced spinal instability/perturbation (i.e. rapid arm raising) [17]. During rapid upper limb movement the transverse abdominis has been shown to become active before the activation or within 50 milliseconds of the primary mover of the upper arm. This feedforward response (defined as occurring within 100 ms before deltoid onset and 50 ms after deltoid onset) is assumed to provide the spinal stability needed to counteract the instability created by the sudden limb movement. In patients with low back injury this feedforward response to sudden limb movement is delayed- the transverse abdominis fails to respond in a feedforward manner [17]. While the influence of viscoelastic creep on muscle function on spine stability properties has been studied extensively, no work has investigated the influence of viscoelastic creep on the superficial transverse abdominis' feedforward response to spine instability induced by sudden arm movement. The aim of this study was to measure the influence of viscoelastic creep on trunk stability as measured by the transverse abdominis' and erector spinae's activation onset timing to sudden rapid arm movement.

## Methods

### Subject Characteristics and Inclusion Criteria

Eleven healthy males and females with no back, hip or upper limb pain or a history of pain within 3 months and low levels of adipose tissue were recruited from a convenience sample of college age (24 to 30 years old) students. Participants read and signed an information and consent form detailing the experiment approved by the Research Ethics Board of the investigating institution (Canadian Memorial Chiropractic College).

### Experimental Procedure

The surface myoelectric activity – and subsequent muscle onsets – of the right transverse abdominis/internal oblique and erector spinae (at L3) was recorded during ballistic raising of the right arm into flexion of 90°. This experimental task occurred immediately before and immediately after a 10 minute static flexion stretch of the lumbar spine-inducing viscoelastic creep.

### EMG Collection and Hardware Characteristics

Disposable bipolar Ag-AgCl disc surface electrodes (Bortec EMG, Calgary, AB) with a diameter of one cm were adhered unilaterally over the muscle groups studied with a fixed centre to centre spacing of 1 cm. EMG electrodes were placed parallel with the muscle fibres, on the skin above the right deltoid, right erector spinae (at the level of L3, 3–4 cm lateral to the spinous process, parallel to the muscle fibres) and right transverse abdominis/internal oblique (approximately 2 cm medial and inferior to the Anterior Superior Iliac Spine, at an angle facing the umbilicus). This site for the transverse abdominis/internal oblique has been shown to be a reliable and valid indicator of measuring the feedforward response of the transverse abdominis with surface EMG electrodes [19]. Raw EMG was amplified between 1000 and 20,000 times depending on the subject. The amplifier had a CMRR of 10,000:1 (Bortec EMG, Calgary AB, Canada). Raw EMG was band pass filtered (10 and 1000 Hz) and A/D converted at 2048 Hz using a NI data acquisition system controlled by Delsys EMGWorks software (Delsys EMG, Boston, MA).

### Sudden arm movement task

Myoelectric activity from the ipsilateral muscles was collected while the subject ballistically raised their right arm to a position parallel to the floor. Participants faced a wall and the experimenter randomly demanded the initiation of shoulder flexion in an attempt to prevent preactivation of the muscles. Five repetitions of this movement occurred over the course of 20 seconds. If the experimenter noticed volitional preactivation (abdominal bracing) in any of the muscle groups the trials were repeated.

### Viscoelastic Creep Stretching Procedure

Subjects warmed up the spine with moderate trunk flexion and extension movements prior to the start of the forward flexion stretch. Subjects were then placed in a seated posture with their knees flexed (between 90–120 degrees) and the hips flexed and externally rotated to allow the soles of the subject's feet to touch one another. The subject then maximally forward flexed their spine and attempted to "hang" on the passive tissues with no attempt to resist the forward bend with muscle activitation. This position was held for 10 minutes.

### EMG Processing & Calculation of Muscle Onsets

The latency time from initiation of deltoid activity to trunk muscle activation was found by determining the time between the onset of deltoid firing and the onset of muscle activation for each of the 2 trunk muscles assessed. The threshold for considering when a muscle was considered "on" occurred when its level of activity was greater than 3 standard deviations of the mean activity found during a 400 millisecond baseline period before the five trials of sudden arm movements occurred. The average latency of the 5 trials from each sudden arm movement session (before and after viscoelastic stretching) was calculated.

After the EMG was collected and stored it was processed using EMG analysis software (EMG Works, Delsys, Boston USA) and a Microsoft Excel spreadsheet program. The raw signal first had its bias removed, was full wave rectified, dual pass filtered at 50 Hz (6^th ^order Butterworth digital filter) and then a moving average (50 ms with an overlap 49 ms) was applied to the data. The moving average ensures that the point in time exceeding the calculated threshold exceeds that threshold, on average, for at least 50 ms in an attempt to avoid false muscle onsets. The data was then exported to an Excel file where the mean and standard deviations of the resting signal were calculated and the point in time that the signal exceeded the threshold value was identified mathematically and confirmed visually. This ensured a visual validation of the muscle onset.

### Statistical Analysis

A paired t-test at the 5% level of significance was used to assess if there is a difference between the average pre-creep and post-creep latency time for the erector spinae and transverse abdominis/internal oblique. Additionally, trials were classified as satisfying the feedforward criteria (muscle onset within 50 ms after the onset of the deltoid and within 100 ms before the onset of deltoid firing). Descriptive statistics of the percentage of trials satisfying the feedforward criteria were calculated. Last, group averages for the different muscles and conditions were found from the trials satisfying the feedforward criteria and paired t-tests were used to determine if statistically significant differences existed between the pre and post creep conditions for those trials satisfying the feedforward criteria.

## Results

**Figure 1:**
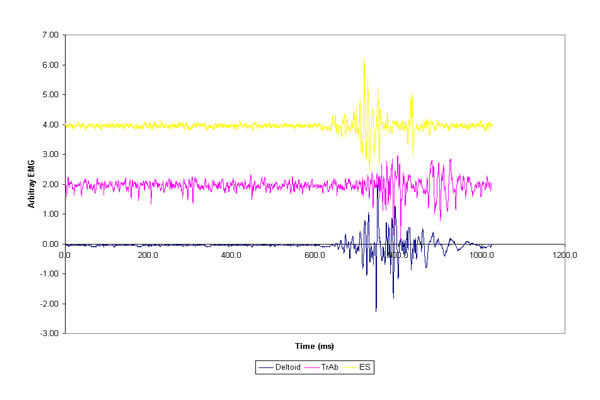
Myoelectric activity of one trial of during a sudden arm raise. A bias to the EMG amplitude has been added to the TransverseAbdominis/Internal Oblique (TrAb) and the Erector Spinae (ESp) for ease of viewing.

The group average for muscle activation delay (after the onset of the deltoid) for transverse abdominus/internal oblique was 59.2 milliseconds (95% Confidence Interval (CI) of 22.5–96.0) for pre-creep and 62.7 (CI = 3 1.5–94.0) for post-creep (positive values indicate that the muscle activation occurred after the deltoid onset). For the erector spinae, delayed muscle activation was 56.9 ms (CI = 1 4.7–99.0) for pre-creep and 69.5 ms (CI = 34.2–104.7) for post-creep. There was no statistical difference in muscle onset latency pre and post the viscoelastic creep protocol. Figure 1 shows a sample (rectified raw EMG) of one trial during the sudden arm raising. The percentage of trials which responded in a feedforward response was also calculated for each muscle and each condition. The transverse abdominis/internal oblique showed a feedforward response during 49% (pre-creep) and 52.7% (post creep) of all trials. The erector spine showed a feedforward response during 60% (pre-creep) and 45.4% (post-creep) of all trials. For individual trial **averages, **only 4/11 participants demonstrated an average transverse abdominis latency response that could be categorized as feedforward for both the pre and post creep conditions. For the erector spinae muscle 5/11 participants showed average feedforward activation pre creep and 6/11 demonstrated an average feedforward response post-creep.

A secondary analysis was done that only included data from trials that satisfied the feedforward criteria. No significant difference was found between the means for both the transverse abdominis and erector spinae pre and post creep protocol. The transverse abdominis group average for those that satisfied the feedforward criteria (n = 8) was 16.7 ms (CI = -1.4–34.9) pre-creep and 26.7 (CI = 13.2–40.2) post creep. The average for the erector spinae pre creep group (n = 11) was 12.0 ms (CI = -5.5–29.5) and post creep was 10.1 ms (CI = -10.5–30.8).

## Discussion

The aim of this study was to determine if prolonged trunk flexion influenced the timing of trunk muscle activation during sudden ballistic arm raises. We found that a forward stretching protocol did not result in a change in the timing of muscle activation onsets for the transverse abdominis/internal oblique and the lower erector spinae as measured from the surface EMG. This finding is in agreement with the work of Granata et al [16] who documented no change in the timing of reflexive muscle activation during random trunk perturbations. The viscoelastic creep stretching procedure used in our study is essentially a means of fatiguing the passive components of the soft tissue posterior elements of the spine. Previous to our study no work had documented the effect of *passive *fatigue on the trunk muscle response to sudden arm raises. However, other researchers have investigated the influence of *active *muscular fatigue on the trunk muscles response to sudden arm raises. Allison and Henry [20] investigated the influence of active muscular fatigue on the trunk muscles *feedforward *response to sudden arm movement and found that following erector spinae fatigue the external oblique muscle onset occurred earlier during trials that exhibited feedforward behaviour. When all trunk muscles (rectus abdominis, external oblique, internal oblique, longissimus and transverse abdominis) were grouped together by side the average onset occurred earlier after active fatigue, while individually there were only trends toward a decrease in the muscle activation onset. In the pilot phase of our study we were unable to consistently find muscle activation in the rectus abdominis and external oblique in response to a sudden arm movement, thus those muscles were excluded from the experiment and the subsequent data collection sessions. This was also seen in the study by Allison and Henry [20] who found inconsistencies in the whether a muscle fired in response to sudden arm raising.

We are uncertain why passive fatigue of spinal tissue did not result in changes in the timing of reflexive or anticipatory trunk muscle onsets yet active fatigue of the trunk muscles appears to result in an earlier onset when muscle firing occurs. One possible explanation for the lack of influence of viscoelastic creep on the feedforward trunk muscle latency may be posture dependent. It is assumed that constant forward bend (the viscoelastic creep stretching protocol) would influence the stiffness of the posterior elements of the spine. In an upright neutral posture ligaments provide very little stiffness in the neutral zone. Therefore a disturbance in ligamentous stiffness in the neutral zone may be unnoticed and have little effect on the neuromuscular response to sudden arm raising because of their limited role in providing stiffness in this neutral upright posture. Testing the anticipatory and reflexive muscle activation to perturbations in non neutral postures may better elucidate the influence of viscoelastic creep on the reflexive and anticipatory behaviour of trunk muscles to perturbations in stability.

A limitation of the study was the large degree of variability across participants and within a single participant across trials. This large variability ensures difficulty in finding statistical significance. While previous research [19] using similar procedures have reported high repeatability across days, within subject variability may be normal as seen in the high between trial variability of this study and the large degree of variability reported in previous studies [20]. This study is also limited by only measuring the surface myoelectric activity. In-dwelling electrode readings of the transverse abdominis and from different sections of the muscle may show different findings.

## Conclusion

Spinal viscoelastic creep did not influence the anticipatory trunk muscle onset timing following ballistic arm movement. A great degree of variability was seen in the onset timing of the trunk muscles and close to half of all trials failed to satisfy the feedforward criteria.

## Authors' contributions

GJL: Conception, design, data collection, data analysis, manuscript preparation.

SS & RM: data collection, data analysis, manuscript preparation.
